# Neurological Findings in Children without Congenital Microcephaly Exposed to Zika Virus in Utero: A Case Series Study

**DOI:** 10.3390/v12111335

**Published:** 2020-11-20

**Authors:** Marília Rosa Abtibol-Bernardino, Lucíola de Fátima Albuquerque de Almeida Peixoto, Geruza Alfaia de Oliveira, Tatiane Freitas de Almeida, Gabriela Ribeiro Ivo Rodrigues, Rodrigo Haruo Otani, Beatriz Caroline Soares Chaves, Cristina de Souza Rodrigues, Anny Beatriz Costa Antony de Andrade, Elijane de Fatima Redivo, Salete Sara Fernandes, Marcia da Costa Castilho, Silvana Gomes Benzecry, Camila Bôtto-Menezes, Flor Ernestina Martinez-Espinosa, Maria das Graças Costa Alecrim

**Affiliations:** 1 Postgraduate Program in Tropical Medicine, University of Amazonas State, Manaus 69040-000, Brazil; peixoto.luciola@gmail.com (L.d.F.A.d.A.P.); beatrizschaves1@gmail.com (B.C.S.C.); fgacristinarodrigues@gmail.com (C.d.S.R.); elijaneredivo@gmail.com (E.d.F.R.); saletesara@yahoo.com.br (S.S.F.); camila.chabm@gmail.com (C.B.-M.); florespinosa@gmail.com (F.E.M.-E.); galecrim.br@gmail.com (M.d.G.C.A.); 2 Tropical Medicine Foundation Dr. Heitor Vieira Dourado, Manaus 69040-000, Brazil; g.alfaia@yahoo.com.br (G.A.d.O.); gabrielaivo@hotmail.com (G.R.I.R.); 3 Department of Medicine, Federal University of Amazonas, Manaus 69067-005, Brazil; tatianefreitas13@gmail.com; 4 Department of Medicine, School of Health Sciences, University of Amazonas State, Manaus 69065-001, Brazil; rod.otani@gmail.com (R.H.O.); sbenzecry@uea.edu.br (S.G.B.); 5 Postgraduate Program in Living Conditions and Health Situations in the Amazon PPGVIDA, Leônidas & Maria Deane Institute, Fiocruz Amazonia, Manaus 69057-070, Brazil; antony.beatriz@gmail.com; 6 Department of Virology, Tropical Medicine Foundation Dr. Heitor Vieira Dourado, Manaus 69040-000, Brazil; mcastilho@fmt.am.gov.br; 7 Department of Malaria, Tropical Medicine Foundation Dr. Heitor Vieira Dourado, Manaus 69040-000, Brazil; 8 Laboratory of Territory, Environment, Health and Sustainability, Leônidas & Maria Deane Institute, Fiocruz Amazonia, Manaus 69057-070, Brazil; 9 Medical Course Coordination, Manaus Metropolitan College/FAMETRO, Manaus 69050-000, Brazil

**Keywords:** arbovirus, Zika virus, infant health, congenital Zika syndrome, coinfection, neurodevelopment, Bayley III, neurologic manifestations, autism, non-microcephalic children

## Abstract

The Zika virus can induce a disruptive sequence in the fetal brain and is manifested mainly by microcephaly. Knowledge gaps still exist as to whether the virus can cause minor disorders that are perceived later on during the first years of life in children who are exposed but are asymptomatic at birth. In this case series, we describe the outcomes related to neurodevelopment through the neurological assessment of 26 non-microcephalic children who had intrauterine exposure to Zika virus. Children were submitted for neurological examinations and Bayley Scales-III (cognition, language, and motor performance). The majority (65.4%) obtained satisfactory performance in neurodevelopment. The most impaired domain was language, with 30.7% impairment. Severe neurological disorders occurred in five children (19.2%) and these were spastic hemiparesis, epilepsy associated with congenital macrocephaly (Zika and human immunodeficiency virus), two cases of autism (one exposed to Zika and *Toxoplasma gondii*) and progressive sensorineural hearing loss (*GJB2* mutation). We concluded that non-microcephalic children with intrauterine exposure to Zika virus, in their majority, had achieved satisfactory performance in all neurodevelopmental domains. One third of the cases had some impairment, but the predominant group had mild alterations, with low occurrence of moderate to severe disorders, similar to other studies in Brazil.

## 1. Introduction

Neurological development is the product of a multifactorial process that depends on the interaction between genetic and environmental aspects, and is influenced by maternal and child nutritional quality, socioeconomic level and stimuli received through experiences [1]. The period that starts at conception and ends at the age of two is the one of greatest development periods of the central nervous system (CNS) and, at the same time, the most vulnerable [2,3].

Various risk factors can disrupt fetal development. Infectious agents can cross the placental barrier and enter the fetal bloodstream causing direct damage by promoting cytotoxic effects, mitotic inhibition or events leading to vascular disruption. Infectious agents can also induce an aggressive reparative response, increasing the lesional area and giving rise to intracranial calcifications [4]. Fetal development disorders, studied together with congenital defects in teratology, are not always evident at birth [5].

Viruses of the *Flaviviridae* family are generally not associated with vertical transmission and are not related to severe fetal alterations [6]. However, after the outbreak of Zika virus (ZIKV) in Brazil, in 2015, severe cases of congenital infection were reported [7,8,9]. Cugola et al. (2016) demonstrated that the Brazilian Zika virus strain is able to cross the placental barrier, infect human progenitor cortical cells and promote cell death by inducing apoptosis and autophagy. Consequently, they observed a reduction in neuronal proliferative zones and disruption of cortical layers [10].

Microcephaly and severe cerebral changes, such as intracranial calcifications, malformations in cortical development, reduction in the volume of cerebral white and gray matter and ventriculomegaly, are the major markers of this new syndrome [9]. Although the most severe cases of central nervous system involvement are already well known, the phenotypic spectrum of congenital Zika virus syndrome (CZVS) has not been fully defined and less severe cases have been described. [11,12,13,14]. Knowledge gaps are still being filled in relation to children exposed to Zika virus during the gestational period who do not present the classic outcomes of CZVS at birth. One of these gaps is as to whether the virus can cause minor disorders perceived later on in the first years of life [15,16]. The surveillance of neuropsychomotor development (NPMD) is important, so that any damage is soon identified and referred for early interventions that will help to develop the child to their full potential [17]. The present study describes neurological outcomes of non-microcephalic children born to pregnant women with laboratory confirmed ZIKV infection.

## 2. Materials and Methods

This study is part of the project named “Epidemiological, clinical, nutrological, virological, histopathological and immunological characteristics of ZIKV infection in pregnant women with acute exanthematous disease and its relationship with microcephaly or possible adverse outcomes in Manaus, Amazonas”. Approval was obtained from the research ethics committee of the Tropical Medicine Foundation Doctor Heitor Vieira (FMT-HVD), under the Certificate of Presentation of Ethical Appreciation (CAAE): 60168216.2.0000.0005, with approval number: 1′806.030, and approval date: 4 November 2016. Parents or legal guardians signed the informed consent form agreeing to the child’s participation.

The year 2015 marked the beginning of the ZIKV epidemic in the city of Manaus with the confirmation of 4418 cases, which involved 500 pregnant women. However, only five cases of microcephaly related to ZIKV were officially reported in the same year [18]. In 2016, 828 pregnant women with exanthematous syndrome were monitored at FMT-HVD, of which 328 (39.6%) received laboratory confirmation of ZIKV infection. After delivery, 78 (23.8%) women continued to be followed-up and allowed pediatric and neurological follow-up of their children. In this cohort, five children (6.4%) were diagnosed with CZVS at birth.

Non-microcephalic children, born between 2016 and 2018, were recruited from this cohort of infants exposed to ZIKV in the intrauterine period, and were followed up at FMT-HVD. Exposure was confirmed by detection of ZIKV in blood or urine samples of mothers during the gestational period using the real-time reverse transcriptase polymerase chain reaction (RT-PCR) technique. The examination was performed in the Central Laboratory of Public Health (LACEN) in Manaus, according to the protocol of Lanciotti et al. (2008) [19]. Considering the great possibility of cross reaction between flavivirus antibodies and the high endemicity of dengue in Brazil, in addition to avoiding invasive procedures in children, research on the serological status was not used in the methodology of this study [11]. The prenatal history of the child was obtained through interviews with the mothers and through secondary data collected from the woman’s pregnancy medical card and records from the electronic medical record at the hospital, which contain the results of serologies performed during routine prenatal control.

The inclusion criteria were children exposed to ZIKV at any time during pregnancy, where infection was confirmed by RT-PCR of maternal samples, even in the presence of other infections in pregnancy, and without microcephaly or other evidence of congenital syndrome at birth. Children older than 42 months and 15 days, the age limit for application of the Bayley Scales of Infant and Toddler Development—Third Edition (BSID-III), were excluded.

Birth data were derived from the child’s medical card and anthropometry was analyzed from the INTERGROWTH-21 charts, considering gestational age at birth, and the child’s sex [20]. The children were followed up by a multidisciplinary team, composed of a pediatrician, a pediatric nutrologist and a pediatric neurologist who performed general clinical evaluations, anthropometry measurements and neurological examinations. For evaluations of preterm growth and development, the chronological age was corrected for prematurity during the first two years of life, according to Babson (1970) [21]. Individual anthropometric measurements were analyzed using the graphs for age and sex, according to the World Health Organization (WHO) standards [22,23]. Head circumference (HC) was considered appropriate for age when the Z score was between −2 SD and +2 SD; microcephaly was deemed when the Z score was less than −2 SD; and macrocephaly when it was greater than +2 SD [22]. The measurements of the head circumference at birth were obtained from secondary data recorded in the child’s medical card, and the neuropediatric specialist measured it again on the day of application of the development scale.

When assessing the neurological aspects of the child, the evolutionary dynamics of the human being in its greatest CNS maturation period must be taken into account. This makes pediatric neurological semiology complex, not only due to the search for pathological locational signs, but also because it involves evaluating adequate patterns of motor, cognitive, language and sensory acquisitions [24]. Following the principle of minimal manipulation of Prechtl, the neurological examination evaluated the alertness of the child, their interaction with the environment, their communicative and social skills, characteristics of crying, posture and attitude. Clinical evaluations of cranial nerves, general mobility, strength and muscle tone, presence of primitive reflexes, deep tendon reflexes and evaluation of the cephalic segment (head circumference, craniofacial proportion, cranial asymmetry, fontanelles and sutures) were also performed [25,26].

Using a tape measure, the cephalic perimeter was measured considering the largest occipito-frontal diameter. Microcephaly and macrocephaly were considered congenital or acquired, whether present at birth or not, and also as either proportional or disproportionate, by relating them to the other anthropometric measurements of the child. Cranial symmetry was verified by biauricular and anteroposterior measurements to obtain the cephalic index of Diament [27]. All the children in the cohort had received neurological examinations at least twice in the first three years of their life.

The instrument used to evaluate neuropsychomotor development was the BSID-III, already validated for the Brazilian population, and applicable to children aged between 16 days and 42 months and 15 days [28,29]. The domains of cognition, language and motor skills were applied by the pediatric neurologist, with expertise in child development, and a physiotherapist, both qualified through specific training in the application and calculation of the score. The results obtained through this scale can be expressed by quantitative and qualitative scores. Total raw scores, scaled scores, composite scores, percentile ranks are examples of quantitative scores. According to the BSID-III technical manual, composite scores are a transformation of a score distribution and have a certain mean and standard deviation. This allows measurements in standard deviation units of the distance of a child’s score from the average. This score ranges from 40 to 160, with 100 being the average performance of a given age group with a standard deviation of 15. Scores of 85 and 115 are, respectively, 1 SD below and above average; 70 and 130 are 2 SD below and above average; 55 and 145 are 3 SD below and above average. Children with a score greater than or equal to 85 (≥−1 SD) are considered to have adequate development. Those between 84 and 70 (<−1 and ≥−2 SD) are considered to have a mild delay or at risk of developmental delay; those with scores between 69 and 55 (<−2 SD and ≥−3 SD) have moderate delay; and those below 55 (<−3 SD) have severe developmental delay. The composite BSID-III score can also be described qualitatively, according to the level of performance of the child. It can be classified as very superior (≥130), superior (120–129), high average (110–119), average (90–109), low average (80–89), borderline (70–79) or extremely low (≤69) [29].

Complementary tests, such as neuroimaging, audiological exams, and electroencephalograms, were requested according to the clinical evolution and demands of each child. These took into consideration the ethical limitations of exposing children to unnecessary sedation or radiation. All children with neurologic alterations on physical examination, or low scores in at least one domain of development, were submitted to an imaging examination of the central nervous system (magnetic resonance or computed tomography). The children with language delay were submitted to a battery of audiological tests, including behavioral hearing assessment, click ABR (auditory brainstem response), air and bone conduction toneburst ABR, tympanometry and otoacoustic emission. Those with suspected epileptic seizures were submitted to electroencephalography.

The application of the BSID-III testing took place in the period from October 2019 to July 2020. Given the scenario of the global pandemic of COVID-19, and in accordance with the government guidelines received, activities were temporarily suspended in the period from March to June 2020. Due to this interruption, some children had already extrapolated the age allowed to perform the test and could not be evaluated by this scale after the return of activities. Some parents refused to participate because they considered their children healthy. In other cases, it was not possible to contact the parents by telephone or at their given address, due to outdated information. Therefore, of the 73 asymptomatic born children of the cohort, 26 were submitted to the test.

## 3. Results

At the time of the application of the BSID-III test, the average age of the 26 children was 37.8 ± 3.95 months (range 25–42 months). Children were predominantly females (14/26; 53.8%). All children were born full-term, 15/26 (57.7%) by cesarean delivery, without report of perinatal asphyxia or hospitalization in a neonatal intensive care unit (ICU).

Data related to demographics, pregnancy, post-natal aspects, and BSID-III results are described in Table 1. The characterization of children regarding the gestational trimester of occurrence of ZIKV infection, gender, the presence of co-infections in pregnancy, age at the application of BSID-III, the head circumference at birth and the current evaluation (z-score), the results of the BSID-III evaluation and the neurological examination are presented in Table 2.

**Figure 1:**
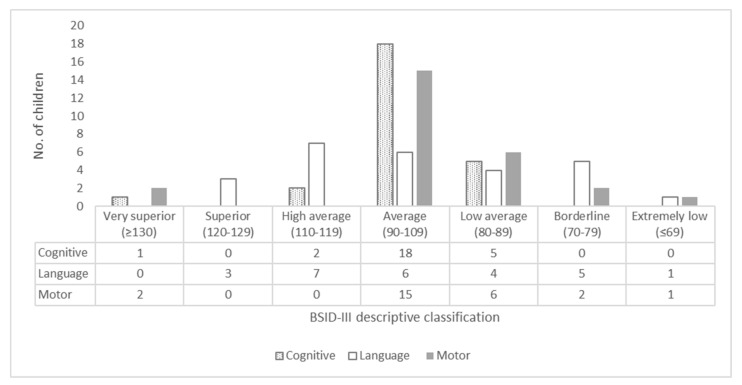
Qualitative description of the composite scores of the cognitive, language and motor domains for 26 non-microcephalic children exposed to the Zika virus in the intrauterine period, Manaus, Amazonas, Brazil.

Of the 26 children who were subjected to the evaluation in the three BSID-III development domains, 17 (65.4%) achieved a satisfactory performance, with a composite score greater than 85 (−1 SD) in all domains. Nine (34.6%) presented abnormal results, in the following distribution: four (15.4%) with one abnormal domain, another four (15.4%) with two abnormal domains and only one (3.8%) with alterations in the three domains. Figure 1 shows the distribution of the performance of each child according to the descriptive classification of the composite score.

The most impaired domain was language, with an alteration in 30.7% (8/26) of children. Of these, 87.5% (7/8) had mild delay (<−1 and ≥−2 SD) and 12.5% (1/8) had severe delay (<−3 SD). The latter was diagnosed with moderate bilateral sensorineural hearing loss, which had occurred through mutation in the GJB2 gene. The second domain with the highest number of alterations was that of motor skills, with a percentage of 19.2% (5/26) of affected children. In this group, 80% (4/5) had mild delay (<−1 and ≥−2 SD), and 20% (1/5) had moderate delay (<−2 SD and ≥−3 SD), and were diagnosed with spastic hemiparesis. The least compromised was the cognitive domain, where only 7.7% (2/26) were affected with mild delay (<−1 and ≥−2 SD). All children with low scores in at least one domain of development or with abnormal neurologic examination were submitted to an imaging examination of the central nervous system, but none showed alterations. The toddlers diagnosed with language delay were subjected to a battery of audiological tests, but only one (1/26; 3.8%) was diagnosed as having suffered hearing loss.

Among the neurological diagnoses detected in this series, we highlight those of five (19.2%) children who presented epilepsy associated with congenital macrocephaly, spastic hemiparesis, as well as the two cases of autism and one of progressive sensorineural hearing loss (caused by mutation in the GJB2 gene). The child diagnosed with epilepsy was a boy who had been exposed to ZIKV in the second trimester and HIV in the first trimester. The mother received triple antiretroviral therapy composed of tenofovir + lamivudine + efavirenz (TDF + 3TC + EFV) and intravenous prophylaxis with azidothymidine (AZT) in peripartum to prevent vertical transmission. She had good adherence to treatment and an undetectable viral load. He was born by cesarean section, received prophylaxis with zidovudine for 4 weeks and did not breastfeed. There was no vertical transmission of retrovirus. He presented congenital disproportionate macrocephaly and, at 21 months, began paroxysmal episodes characterized by behavioral arrest, skin pallor and loss of tone, which was adequately controlled with sodium valproate. A transfontanellar ultrasound performed at two months of age suggested discrete dilatation of the third ventricle, however a brain magnetic resonance imaging (MRI) at 36 months did not show any alterations.

In relation to other neurological diagnoses detected in this series, one child (female) presented a delay in gait acquisition and was detected as having mild spastic hemiparesis. She was diagnosed, after the first year of life, with cerebral palsy and classified as level 1 in the gross motor function classification system (GMFCS*).* One child (male) with autism was exposed to ZIKV in the first gestational trimester and coinfected with *T. gondii.* The mother received treatment with sulfadiazine, pyrimethamine and folinic acid during pregnancy. The boy presented low performance in the language domain in the BSID-III evaluation, with marked irritability, impairment in joint attention, repetitive and stereotyped behavior, and inflexibility to routines. Another child was exposed only to ZIKV, and also presented difficulty in reciprocal social communication and in social interaction, as well as restricted and repetitive patterns of behavior, interests or activities. The girl with sensorineural hearing loss continued to present speech delay, impairment in joint attention, echolalia, and repetitive and stereotyped behavior. The genetic panel report for hearing loss has detected a mutation in the GJB2 gene, ruling out the possibility of association with intrauterine exposure to the Zika virus. The characteristics related to neurological examination and the performance in the BSID-III evaluation are shown in Table 3.

## 4. Discussion

The ZIKV, a flavivirus transmitted mainly through the bite of arthropods of the genus *Aedes,* was considered harmless, since it appeared to cause only mild symptoms in those infected [30,31]. In 2015, however, it became a reason for international attention after the recognition of the severe effects of prenatal exposure on newborns [32]. It was discovered that ZIKV induces a disruptive sequence in the fetal brain, with microcephaly as its main marker [7,8,9]. Since then, this virus has been considered as a new member of the following group of pathogens: *Toxoplasma gondii*; others; rubella; cytomegalovirus; herpes simplex (TORCH) for being able to generate severe damage to the fetus through vertical transmission of infection [6,16]. Currently, efforts are aimed at understanding the outcomes in children who were exposed in the antenatal period, but who were born without the congenital stigmas [12,13,14].

This series of cases shows that 65.4% of the fetuses exposed to ZIKV in pregnancy, born without microcephaly, presented satisfactory outcomes in neuropsychomotor development. There was a predominance of mild delays (26.9%), followed by moderate motor delays (3.8%) and severe language delays (3.8%).

Regarding the qualitative classification of composite scores of domains evaluated by BSID-III, there was a predominance of scores in the “average” range, between 90 and 109 points, which are similar to the results obtained in a cohort of children in the city of Tanguará da Serra, Mato-Grosso, Brazil. In this study, Gerzson et al. (2019) evaluated NPMD in 17 non-microcephalic children older than 18 months using the same instrument. They concluded that, in the fetuses exposed to ZIKV during pregnancy, the majority of the normocephalic births presented typical development [13]. Faiçal et al. (2019), using BSID-III, evaluated 29 children from the city of Salvador, Bahia, Brazil with a mean age of 18.2 months. They detected alterations in 34.5% (10/29) of children in at least one of the three domains. Mild delays were the most frequent, in 27.7% (8/29), followed by 3.4% (1/29) with moderate delays and also 3.4% (1/29) with severe delays, both were in language [12]. Using BSID-III, Nielsen-Saines et al. (2019) followed-up a prospective cohort of 146 non-microcephalic children, aged between 7 and 32 months, who had suffered antenatal exposure. They found satisfactory results in 60% of children. Among those with altered results, mild delays were also the most frequent in 28%, with 12% of the children presenting scores below 70 (<−2 SD) [33]. Cranston et al. (2020) described neurodevelopmental outcomes using BSID-III in a cohort of 112 normocephalic children aged between 6 and 42 months that had had antenatal exposure to ZIKV and found adequate NPMD in 64.3%. Among those affected, children with mild impairment or considered at risk of developmental delay prevailed in 26.8% of the group. Only 8.9% had scores lower than −2 SD, with significant delay. The mean performance for each domain of development were as follows: 99.91 for cognition; 95.43 for motricity; 89.12 for language [14].

Among those with alterations (approximately 35%), the predominance of the group with mild alterations or at risk of developmental delay is highlighted. The most impaired domain was language, which is similar to the three aforementioned studies [12,14,33]. In the general population, the percentage of children with delays in the early stages of speech development is approximately 16%. Of these, half can persist with losses that may have repercussions throughout life [34]. Maternal ZIKV infection in first gestational trimester has been associated with a higher risk of adverse outcomes on the fetus [16,35]. In the present study, impairment occurred more frequently in the second trimester.

Pathogens of the TORCH group are known to be teratogenic [4,6,16]. Although sometimes the teratogenic agent is aggressively harmful to the fetus, in most cases, it may not interfere with fetal development or cause harm [4]. We describe two cases of coinfection with dengue virus (DENV), one with herpes simplex virus (HSV), one with human immunodeficiency virus (HIV), and another with *Toxoplasma gondii*. Only in those coinfected with DENV were no changes in neurological outcomes observed, as detailed below.

Exposure to DENV, a virus of the *Flaviviridae* family, can most often cause prematurity and low birth weight [36]. The two children who had been exposed to ZIKV and DENV did not present perinatal complications or alterations in neurodevelopment. The HC was normocephalic and the mean BSID-III scores in the domains of cognition, language and motor were 100, 109 and 105, respectively.

Microcephaly and other severe changes can be caused by viruses of the *Herpesviridae* family, such as cytomegalovirus (CMV), herpes simplex (HSV) and varicella zoster (VZV), but more rarely since transplacental infection occurs less frequently [16]. The child who suffered exposure to ZIKV and HSV presented a head circumference at the lower limit of normality at birth (−2 SD) and low scores in the domains of cognition and language of 80 and 83, respectively. A Brazilian cohort study described adverse effects in 787 children exposed to HIV and antiretroviral therapy in the pre and neonatal period. It highlighted, as the most frequent outcomes, changes in liver function (36%), anemia (25.7%), low birth weight (22.5%), prematurity (21.7%), small newborn size for gestational age (18%), birth defects (10%) and thrombocytopenia (3.6%). Congenital CNS defects were relatively low, being present in only 2.9% of children [37]. In the present study, the child exposed to HIV and ZIKV during pregnancy also received prenatal and post-natal prophylactic treatment. There was no vertical transmission, verified through negative serologies for HIV after 18 months of age. At birth, disproportionate macrocephaly was detected, still present at the time of the NPMD evaluation. In the BSID-III evaluation, the child presented low scores in the domains of language and motor skills, as well as irritability and impairment in joint attention. At 21 months, he started to suffer from epilepsy, which was well controlled with the use of sodium valproate. Many factors may be related to the unfavorable neurological outcome. Although ZIKV is a dangerous teratogenic agent, there is evidence that exposure to HIV (without infection) may also be associated with impaired neurological development when these children are compared to unexposed children [38,39,40]. The activation of immune response has been demonstrated in fetuses and infants exposed to HIV, and can be a trigger for the production of cytokines that can act directly on cellular migration and axonal growth, and thus have an impact on global brain development [39]. Other factors, such as the activation of the maternal inflammatory process, toxicity of antiretrovirals, environmental enteric dysfunction (a subclinical disorder of the small intestine with atrophy of the villi, damage to the function of the intestinal barrier and microbial translocation, which promotes systemic inflammation), early-life programming influenced by the uterine environment (epigenetics), malnutrition and toxic stress, may also be related to neurological changes [38,40]. Two cases of autism were confirmed, one was exposed exclusively to ZIKV, and the other had coinfection with *T. gondii*. Vianna et al. (2019) suggest the possibility of intrauterine ZIKV infection predisposing children to the occurrence of autistic spectrum disorder (ASD) through the neuro-immunomodulation model [41]. This is based on the fact that, in the presence of an exacerbated inflammatory response to viral infection, pro-inflammatory cytokines, such as interleukin-6 (IL6) and tumor necrosis factor alpha (TNF-α), are released at high levels. IL-6 has been described as a major factor involved in the genesis of ASD, and thus predisposing the occurrence of the disorder [41,42]. On the other hand, studies have suggested that fetal infection by *T. gondii* can also significantly alter the developing brain and may manifest itself in alterations in the first years of life [43,44]. Nielsen-Saines (2019) reported the occurrence of three cases of ASD in a cohort of 216 children exposed to ZIKV in the intrauterine period, which equates to a ratio of 1:72. However, this incidence is not higher than that of the general population—of 1:54 children—according to the Centers for Disease Control and Prevention (CDC) [33,45]. In this series, we report the case of a child that was exposed to ZIKV during the third gestational trimester, normocephalic, without a history of perinatal complications, and presented neonatal hearing screening without alterations. The girl evolved with specific language delay and was diagnosed with moderate sensorineural hearing loss at 36 months of age. There were no alterations in brain magnetic resonance or mastoid computed tomography. She obtained scores in BSID-III that were low in the three domains, the language domain being markedly impaired, with severe delay. On neurological examination, she presented difficulty in sustaining eye contact and impairment in joint attention. It is known that viral infections can occur with congenital or acquired hearing loss, most commonly of the sensorineural type. Various mechanisms may be involved, from direct injury to inner ear structures to the induction of host-exacerbated immune response [46]. However, this girl was diagnosed with a mutation in the GJB2 gene, ruling out the possibility of association with intrauterine exposure to the Zika virus. Studies have reported the occurrence of sensorineural hearing loss in children with CZVS [47,48]. Nevertheless, in most children with microcephaly, there was no evidence of damage to the structures of the inner ear, and alterations in the brain stem and cortical level are more likely to be responsible for such an injury [46]. Leal et al. (2016) performed a retrospective evaluation of 70 children between 0–10 months with microcephaly associated with CZVS. Of these, four (6%) presented sensorineural hearing loss [47]. Cranston et al. (2020) reported the occurrence of hearing alterations in 9.9% (14/141) of non-microcephalic children [14]. The need for studies to clarify the prognosis of hearing health of exposed children without severe CNS involvement is thus highlighted. Furthermore, the importance of undertaking genetic evaluation of hearing loss must not be ignored.

In relation to the neurological diagnoses detected in this series, the presence of irritability and impairment in joint attention stand out as the most frequent. Considering the importance of early diagnosis in the context of autistic spectrum disorder and the search for possible causative agents, markers for early detection of ASD have been studied. Joint attention is the ability to share attention with others by pointing, showing, and coordinating the gaze and the objects or people during an interaction. The impairment in joint attention is one of the markers for the early detection of ASD [49]. Persistent irritability without apparent cause is also a warning sign for problems in psychosocial development. Although it is a frequent symptom among children, irritability can be part of the clinical presentation of numerous disorders and is a predictor of long-term psychopathology [50]. One child presented mild spastic hemiparesis and received a diagnosis of cerebral palsy after the first year of life, which was classified using the GMFCS at level I [51]. This highlights the need for attention regarding children exposed to infections, especially ZIKV, in the prenatal period, since some subtle changes become evident in the course of the maturational evolution of the CNS. One cohort with a higher number of non-microcephalic children exposed to ZIKV showed neurological alterations in 68.1% of the cases [14]. When analyzing the neurological outcomes of the child, not only quantitative but qualitative analyses should be used, considering the dynamic, evolutionary, and maturational aspects of the brain in its period of greatest development [26].

The small sample size in this study prevents a more robust statistical analysis. Despite regional differences, both studies, with small and large samples, presented results similar to that of this case series, with more than 60% of children with adequate neuro-psychomotor development when using the same instrument. The lack of a reliable control group (one in which children had not been exposed to ZIKV) in these studies is a limitation for establishing the risk to the population, and it cannot confirm that these results would be different in the non-exposed population. The accuracy of the tests is impaired by the possibility of cross-reaction with antibodies against dengue and the fact that 50% of mothers may present asymptomatic cases not diagnosed in pregnancy [11].

## 5. Conclusions

Currently, the severe aspects of Zika virus involvement on the fetus are well known. However, less severe manifestations in newborns without signs of congenital ZIKV impairment are also important and should be studied more. The results of this series show neurodevelopmental outcomes similar to those of other case series conducted in Brazil. We concluded that non-microcephalic children with intrauterine exposure to ZIKV, in their majority, had achieved satisfactory performance in all neurodevelopmental domains. One third of the cases had some impairment, but the predominant group had mild alterations, with low occurrence of moderate to severe disorders. The tragedy of the ZIKV epidemic made the invisible child visible by exposing the needs of children with developmental disabilities, often neglected by public health policies. It has highlighted the need to invest in quality childcare, aimed not only at children with visibly compromised neurological development, but aimed at all children, since the subtlety of signs and symptoms may go unnoticed in the window of opportunity. Early intervention in children with developmental delays can prevent the progression of damage and promote the overcoming of such delays, and thus positively impact their prognosis throughout life.

## Figures and Tables

**Table 1:** Demographics, pregnancy, post-natal aspects, and Bayley Scales of Infant and Toddler Development—Third Edition (BSID-III) results of 26 non-microcephalic children exposed to the Zika virus in the intrauterine period, Manaus, Amazonas, Brazil.

Demographics, Pregnancy and Post-Natal Aspects	Typical NPMD *n* = 17	Delayed NPMD *n* = 9
Mother’s age mean ± SD (range)	28 ± 5.4 (20–30)	29.2 ± 8.18 (17–40)
Maternal alcohol intake, *n* (%)	1 (5.9%)	1 (11.1%)
Maternal tobacco intake, *n* (%)	1 (5.9%)	0 (0.0%)
Maternal hypertensive disorder, *n* (%)	3 (17.6%)	1 (11.1%)
Bleeding in the first gestational trimester, *n* (%)	0 (0.0%)	2 (22.2%)
Maternal depression, *n* (%)	0 (0.0%)	1 (11.1%)
Trimester of infection		
First, *n* (%)	5 (29.4%)	2 (22.2%)
Second, *n* (%)	6 (35.3%)	6 (66.6%)
Third, *n* (%)	6 (35.3%)	1 (11.1%)
Coinfection, *n* (%)	2 (11.7%)	3 (33.3%)
Gender		
Male, *n* (%)	6 (35.3%)	6 (66.6%)
Female, *n* (%)	11 (64.7%)	3 (33.3%)
Apgar 5′ (mean ± SD)	9.7 ± 0.6	9.8 ± 0.3
Birthweight classification		
SGA, *n* (%)	1 (5.9%)	2 (22.2%)
AGA, *n* (%)	16 (94.1%)	7 (77.7%)
LGA, *n* (%)	0 (0.0%)	0 (0.0%)
Dysmorphisms, *n* (%)	2 (11.7%)	4 (44.4%)
Double misplaced hair whorls, *n* (%)	0 (0.0%)	2 (22.2%)
Epicanthus, *n* (%)	2 (11.7%)	1 (11.1%)
Flattened nasal base, *n* (%)	0 (0.0%)	1 (11.1%)
Hyperchromic spots, *n* (%)	0 (0.0%)	1 (11.1%)
Change in iris coloration, *n* (%)	0 (0.0%)	1 (11.1%)
Neonatal jaundice, *n* (%)	5 (29.4%)	2 (22.2%)
Neonatal sepsis, *n* (%)	1 (5.9%)	0 (0.0%)
Ototoxic antibiotics in neonatal period, *n* (%)	1 (5.9%)	0 (0.0%)
Exclusive breastfeeding at 6 months, *n* (%)	17 (100%)	8 (88.8%)

NPMD: neuropsychomotor development; n: number; SD: standard deviation; SGA: small for gestational age; AGA: appropriate for gestational age; LGA: large for gestational age.

**Table 2:** Gestational trimester of occurrence of Zika virus (ZIKV) infection, gender, the presence of coinfections in pregnancy, age at the application of BSID-III, the head circumference at birth and the current evaluation (z-score), the results of the BSID-III evaluation and the neurological examination of 26 non-microcephalic children exposed to the Zika virus in the intrauterine period, Manaus, Amazonas, Brazil.

	ID	Gen	Coinfection	Current Age (Months)	HC at Birth (Z-Score)	Current HC (Z-Score)	HC Classification	Cognition Score (IC 95%)	Language Score (IC 95%)	Motor Skills SCORE (IC 95%)	BSID-III Result	Neurological Examination
1° GT ZIKV	1	F	No	-	−0.83	−0.47	Normo	105 (97–113)	106 (98–113)	97 (90–105)	Normal	Adequate
	2	F	No	40	0.08	1.07	Normo	115 (106–122)	124 (115–130)	133 (123–138)	Normal	Adequate
	3	F	No	41	−1.28	0.6	Normo	100 (92–108)	115 (107–121)	103 (95–110)	Normal	Adequate
	4	F	No	42	−0.78	−0.07	Normo	95 (87–103)	91 (84–99)	133 (123–138)	Normal	Adequate
	5	F	No	41	−0.02	−1.03	Normo	85 (78–94)	79 (73–88)	64 (59–75)	Delayed	Altered
	6	M	Dengue	33	1.24	0.81	Normo	100 (92–108)	112 (104–118)	103 (95–110)	Normal	Adequate
	7	M	Toxoplasm.	40	0.08	−1.14	Normo	85 (78–94)	71 (66–80)	85 (79–94)	Delayed	Altered
Mean	-	-	-	38.43	−0.22	−0.03	-	97.86	99.71	102.57	-	-
2° GT ZIKV	8	M	No	25	−0.78	−0.08	Normo	105 (97–113)	77 (71–86)	91 (84–99)	Delayed	Adequate
	9	F	No	34	2	−1.28	Normo	95 (87–103)	89 (83–97)	85 (79–94)	Normal	Altered
	10	M	No	35	−0.63	−1.01	Normo	105 (97–113)	112 (104–118)	107 (99–114)	Normal	Adequate
	11	F	No	36	−1.62	−1.67	Normo	100 (92–108)	91 (84–99)	82 (76–91)	Delayed	Adequate
	12	F	No	37	1.11	1.18	Normo	100 (92–108)	115 (107–121)	107 (99–114)	Normal	Adequate
	13	M	No	40	0.08	−0.98	Normo	90 (83–99)	77 (71–86)	85 (79–94)	Delayed	Altered
	14	M	No	40	2.16	1.94	Macro resolved	85 (78–94)	71 (66–80)	76 (70–86)	Delayed	Altered
	15	F	No	41	0.22	0.69	Normo	105 (97–113)	91 (84–99)	97 (90–105)	Normal	Adequate
	16	F	No	41	2.68	1.63	Macro resolved	115 (106–122)	115 (107–121)	103 (95–110)	Normal	Adequate
	17	M	HIV	34	2.56	2.21	Macro maintained	90 (83–99)	83 (77–91)	73 (68–83)	Delayed	Altered
	18	M	Herpes	38	−2	−0.16	Normo	80 (74–90)	83 (77–91)	85 (79–94)	Delayed	Adequate
	19	F	Dengue	41	1.92	0.95	Normo	100 (92–108)	106 (98–113)	107 (99–114)	Adequate	Adequate
Mean	-	-	-	36.83	0.64	0.29	-	98	95.6	93	-	-
3° GT ZIKV	20	F	No	36	1.94	1.11	Normo	105 (97–113)	109 (101–116)	103 (95–110)	Adequate	Adequate
	21	M	No	37	1.43	1.41	Normo	105 (97–113)	124 (115–130)	103 (95–110)	Adequate	Adequate
	22	M	No	38	−0.27	0.11	Normo	90 (83–99)	118 (110–124)	91 (84–99)	Adequate	Altered
	23	F	No	38	1.94	0.41	Normo	105 (97–113)	121 (112–127)	100 (92–108)	Adequate	Adequate
	24	F	No	41	1.38	0.41	Normo	80 (74–90)	53 (49–64)	82 (76–91)	Delayed	Altered
	25	M	No	41	−0.78	−0.25	Normo	130 (119–135)	89 (83–97)	107 (99–114)	Adequate	Adequate
	26	M	No	42	−0.78	1.02	Normo	90 (83–99)	115 (107–121)	97 (90–105)	Adequate	Adequate
Mean	-	-	-	39.00	0.69	0.60	-	100.71	104.14	97.57	-	-
Total Mean	-	-	-	37.85	0.43	0.29	-	98.46	97.58	96.12	-	-

Id: identification; Gen: gender; HC: head circumference; GT: gestational trimester; ZIKV: Zika virus; CI 95%: confidence index of 95%.

**Table 3:** Characteristics related to neurological examination and the performance in the BSID-III evaluation for 26 non-microcephalic children exposed to the Zika virus in the intrauterine period, Manaus, Amazonas Brazil.

Neurologic Examination, No. (%)	Typical NPMD 17 (65.4%)	Delayed NPMD 9 (34.6%)
Adequate 18 (69.3%)	14 (82.3%)	4 (44.4%)
Altered 8 (30.7%)		
Irritability	1 (5.8%)	4 (44.4%)
Impairment in joint attention	1 (5.8%)	6 (66.6%)
Psychomotor agitation	0 (0.0%)	3 (33.3%)
Congenital macrocephaly		
Resolved in the first year of age	1 (5.8%)	1 (11.1%)
Maintained	0 (0.0%)	1 (11.1%)
Motor deficit (hemiparesis)	0 (0.0%)	1 (11.1%)
Impaired muscle tone		
Spastic hypertonia	0 (0.0%)	1 (11.1%)
Hypotonia	1 (5.8%)	0 (0.0%)
Increased deep tendon reflexes	0 (0.0%)	1 (11.1%)

No.: number of children.
